# Deciphering Hepatic Dilemmas: A Case of Acute Hepatitis Following Malaria Treatment

**DOI:** 10.1155/crgm/9995249

**Published:** 2025-12-28

**Authors:** Sydney Lovrien, Erin Quist, Dayana Maita Coronel, Ashwani K. Singal, Chencheng Xie

**Affiliations:** ^1^ Department of Internal Medicine, Sanford School of Medicine, University of South Dakota, Sioux Falls, South Dakota, USA, sdstate.edu; ^2^ Department of Pathology, Sanford School of Medicine, University of South Dakota, Sioux Falls, South Dakota, USA, sdstate.edu; ^3^ Division of Pathology, Avera McKennan Hospital & University Health Center, Sioux Falls, South Dakota, USA, avera.org; ^4^ Division of Infection, Avera McKennan Hospital & University Health Center, Sioux Falls, South Dakota, USA, avera.org; ^5^ Department of Medicine, University of Louisville School of Medicine, Louisville, Kentucky, USA, louisville.edu; ^6^ Division of Gastroenterology and Hepatology, Roy J. and Lucille A. Carver College of Medicine, University of Iowa, Iowa City, Iowa, USA, uiowa.edu

**Keywords:** drug-induced liver injury, hepatitis, malaria

## Abstract

Malaria can be a life‐threatening disease, but it rarely presents with acute hepatitis. This case reports the medical course of a 55‐year‐old African woman afflicted with *P. falciparum* malaria, recently treated in Cameroon, who presented with jaundice and transaminitis. Her liver biopsy indicated cholestatic hepatitis. Her diagnosis was complicated by the fact that only one of four follow‐up thick and thin blood smears was positive for *P. falciparum.* After more than 17 months of follow‐up, her liver enzymes and bilirubin returned to baseline without further malaria treatment, and her findings were attributed to drug‐induced liver injury caused by antimalarial medications.

## 1. Introduction

This is a unique case where an individual had a confirmed *P. falciparum* infection that was thought to be cleared by antimalarial medication (substantiated by multiple negative thick and thin blood smears) who continued to have jaundice, transaminitis, abdominal pain, nausea, and vomiting. This report aims to describe and explain the diagnostic challenges for a case of liver injury that occurred after recent antimalarial treatment.

## 2. Case Presentation

A 55‐year‐old African female presented to the emergency department with complaints of jaundice, fatigue, extremity swelling, generalized abdominal discomfort, nausea, vomiting, diarrhea, decreased oral intake, and a 20‐pound weight loss secondary to malaria. She traveled from Cameroon, where she was diagnosed with malaria. *P. falciparum* species, which was identified on blood smears. The patient had been hospitalized for 2 weeks in Cameroon and was treated with two unspecified antimalarial agents, oral and intravenous, 2‐3 weeks prior to this admission. The follow‐up smears checked in Cameroon were reported as negative for parasites, and the patient was subsequently discharged. She returned to the United States and was readmitted due to symptom recurrence.

The patient’s past medical history was significant for latent tuberculosis (treated), microcytic anemia, and hypertension. She reports no allergies or prior surgical history. She has a family history of sickle cell disease, and several family members have previously contracted malaria. Hemoglobin electrophoresis confirmed sickle cell trait in the patient.

The patient remained afebrile throughout the course of the illness. She was visibly jaundiced with a mildly tender abdomen. A 3/6 systolic murmur and trace lower extremity edema were appreciated on the exam. The remainder of her physical exam was unremarkable.

**Figure 1:**
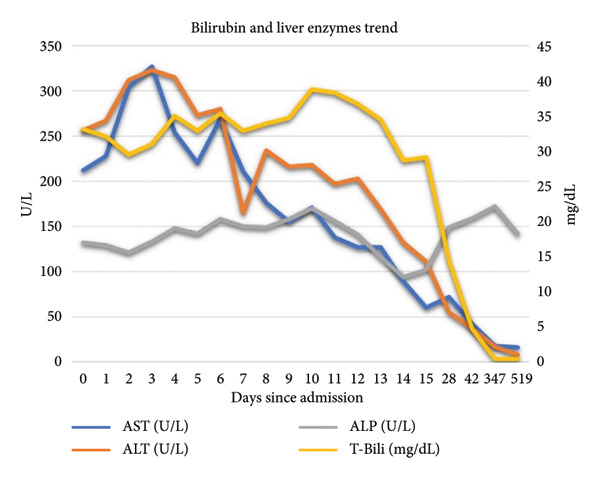
Trend of bilirubin and liver enzymes. ALT‐alanine transaminase, AST‐aspartate aminotransferase. ALP‐alkaline phosphatase. T‐Bili‐total bilirubin*.* AST normal range: 13–39 U/L. ALT normal range: 5–25 U/L. ALP normal range: 34–104 U/L. T‐Bili normal range: 0.3–1.0 mg/dL.

The patient’s initial liver enzymes were uniformly elevated, with a total bilirubin of 33.1 mg/dL, predominantly conjugated hyperbilirubinemia with 22.7 mg/dL composed of direct bilirubin. AST was elevated at 212 U/L, ALT at 256 U/L, and alkaline phosphatase at 123 U/L. These values were trended throughout admission, as seen in Table 1 and Figure 1.

**Table 1:** Summary of bilirubin and liver enzymes trend.

2‐3 weeks before admission	Diagnosed with malaria *P. falciparum*, received antimalarial agents, hospitalized for 2 weeks, and discharged				
Days since admission	T‐Bili (reference: 0.3–1.0 mg/dL)	AST (reference: 13–39 U/L)	ALT (reference: 5–25 U/L)	ALP (reference: 34–104 U/L)	
0	33.1	212	256	132	Admission
1	32.1	228	267	129	
2	29.5	304	312	121	
3	31	327	323	133	
4	35	254	315	148	
5	32.9	220	273	142	
6	35.4	269	280	158	
7	32.9	211	165	150	Liver biopsy
8	33.9	176	234	149	
9	34.7	155	216	158	
10	38.8	171	218	170	
11	38.3	138	197	156	
12	36.7	127	203	141	
13	34.5	127	169	116	
14	28.7	89	132	94	
15	29.1	60	111	102	Discharge
28	14.4	72	55	149	Follow up
42	4.7	42	37	159	Follow up
347	0.4	18	17	172	Follow up
519	0.4	16	8	142	Follow up

Abbreviations: ALP, alkaline phosphatase; ALT, alanine transaminase; AST, aspartate aminotransferase; T‐Bili, total bilirubin.

INR was mildly elevated up to 1.9 in the first week and returned to the normal range in the second week of hospitalization. She was never encephalopathic and did not meet the criteria of acute liver failure. Serologic workups were negative for acute infection of hepatitis A, C, and E. Hepatitis B core antibody was positive; however, hepatitis B surface antigen, surface antibody, and DNA quantitative were negative. Therefore, she is unlikely to have active acute or chronic hepatitis B infection. HIV and adenovirus were negative. Autoimmune hepatitis markers were unremarkable, apart from an elevated ANA (1: 320) and mildly elevated IgG (1746 mg/dL). Ferritin was elevated at 1133 ng/mL. Ceruloplasmin and alpha‐1‐antitrypsin were within the normal range. Thyroid peroxidase antibodies were incidentally found to be elevated, and exophthalmos was observed. She was subsequently diagnosed with Graves’ disease. However, without evidence of a toxic thyroid storm, methimazole and propranolol were initiated. A CT of the abdomen showed splenomegaly, calcifications within the liver, and scattered lymphadenopathy within the inguinal and external iliac chain lymph nodes.

**Figure 2:**
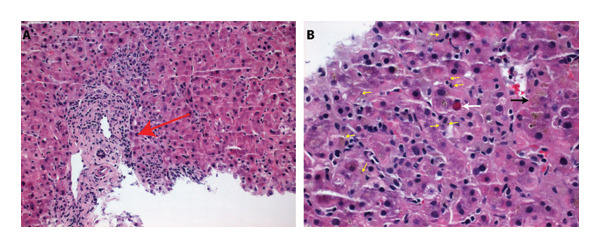
Liver biopsy reveals cholestatic hepatitis. (A) Mild portal inflammation with preserved, unremarkable portal components (red arrow) (10x) and (B) granular pigment (yellow arrows), individual hepatocyte necrosis with acidophil body (white arrow); lobules with cholestasis (black arrow) (40x).

A random liver biopsy was obtained and showed rare portal triads with a mild inflammatory infiltrate composed of lymphocytes, occasional plasma cells, and rare neutrophils. The lobules were composed of benign‐appearing hepatocytes with scattered inflammatory cells. Small clusters of swollen hepatocytes with focal hepatocyte necrosis (acidophil bodies) were noted. The black‐brown finely granular pigment was noted scattered throughout the lobules (Figure 2). Granulomata, pericellular fibrosis, portal fibrosis, increased iron deposition, and alpha‐1‐antitrypsin type inclusions were not identified on routine H&E stains or with the use of special stains.

The patient’s symptoms and laboratory results improved during her 2‐week hospitalization course. Three pairs of thin and thick blood smears (CDC diagnostic standard methods) were reviewed during her hospitalization without evidence of parasitic organisms. However, 1 week after discharge, the last pair of thick and thin smears both demonstrated *P. falciparum* organisms, posing confounding findings and increasing diagnostic challenges. After consulting the CDC, malaria treatment was not recommended, given that no parasite ring forms were visualized on the smear. Additionally, inconsistent smear results and parasitemia < 1% further supported not starting antimalarial treatment. The patient’s clinical course has been followed for 17 months, and liver enzyme and bilirubin levels improved consistently after discharge. Her bilirubin and liver enzymes were found to have returned to baseline at her 11‐month follow‐up visit.

## 3. Discussion

In 2020, malaria was responsible for approximately 241 million clinical cases and led to 627,000 fatalities [1]. This disease stands as a major global public health challenge, particularly in many developing nations where it remains a primary cause of morbidity and mortality. Close to half of the global population resides in regions susceptible to malaria transmission. The WHO African Region bore the brunt and accounted for an estimated 95% of all malaria‐related deaths in 2020 [1]. The predominant parasite responsible for these cases is *P. falciparum*, transmitted through the *Anopheles mosquito*. This particular species is notorious for causing severe malaria infections, which can result in death [1].

Malarial infections are mainly characterized by a recurrent cycle of fevers and chills absent in this patient [2]. Furthermore, malaria rarely presents with acute hepatitis. There is high mortality and poor prognosis for patients with malaria hepatitis, with an estimated 40% mortality [3]. Once malaria involves liver injury, the parasite could cause erythrocytes to adhere to the endothelial walls of liver capillaries, which could block sinusoids and alter the blood flow toward the liver and eventually lead to complications, including hepatic encephalopathy, septicemia, renal failure, and respiratory failure [3, 4]. However, these complications were absent in the patient, and she was asymptomatic for most of the following admission and after discharge.

While the liver biopsy revealed scattered individual hepatocyte necrosis, cholestasis, and black‐brown granular pigment suggestive of “malarial pigment,” sinusoidal red blood cells and malarial organisms presented in some malaria hepatitis cases were not identified in this case [5]. Of note, in our biopsy, the dark granular “malarial pigment” is best interpreted as hemozoin within hepatic macrophages (Kupffer cells), a known sequestration site during and after malarial infection. The hemozoin can be retained in Kupffer cells for months after parasites have cleared. In vivo mouse studies show quantifiable pigment up to 6.5 months post‐treatment [6]. Although direct longitudinal human liver data are scarce, this tissue‐level pigment persistence is well recognized, and it is interpreted that pigment alone reflects recent infection, not necessarily indicating an active/acute infection. Ultimately, the biopsy demonstrated a cholestatic pattern of injury. The differential diagnosis for this histologic pattern includes drug‐induced liver injury, infection, and obstruction [7]. Diagnostic histologic features of autoimmune hepatitis are not seen in this case. There is no evidence of ductopenia or bile duct injury, thus it is unlikely to have primary biliary cholangitis, either. Graves’ disease usually presents with ALP being the most common liver enzyme elevated during thyrotoxicosis [8]. However, this patient lacks thyrotoxicosis and has been treated timely; thus, we believe it is an accidental finding and unlikely to explain her clinical and pathological presentation.

Crucially, the patient did not undergo a second round of malaria treatment following her admission. Yet, she exhibited a rapid improvement in liver enzyme levels through supportive care alone, with a complete resolution observed over a few months. Unfortunately, we were unable to ascertain the specific type and duration of the antimalarial treatment she received in Cameroon. Many antimalarial medications have been documented as potential causative agents of liver injury. For example, amodiaquine is commonly used in combination with other agents for therapy of chloroquine‐resistant *P. falciparum*, and it could cause severe hepatitis and agranulocytosis. Pyrimethamine and sulfadoxine remain a choice of combination with other agents in the treatment of *P. falciparum* malaria, but the hepatotoxicity has raised concerns. Another combination example of atovaquone with proguanil is used for the prevention and treatment of chloroquine‐resistant *P. falciparum*, and this combination has been linked to transient serum enzyme elevations as well. Artemisinin derivatives, including artesunate, artemisinin, dihydroartemisinin, artemether, and arteether, have been widely used in combination with other agents for the therapy of malaria throughout the world. However, many of the artemisinin derivatives have been reported with idiosyncratic liver injury cases [9]. It needs to be noted that while the majority of cases can be effectively treated and cured, latent or relapsing *P. falciparum* malaria could occur years following initial exposure despite a negative smear. The mechanism of this prolonged persistence requires further investigation. The current hypothesis relates to the ability of *P. falciparum* parasites to survive and remain dormant in the body for extended periods, especially after incomplete treatment [10].

There is some overlap in clinical presentation and pathology findings between malaria‐related hepatitis and DILI. In our case, we have a prolonged follow‐up, and the conclusion rests on the clinical presentation associated with medication exposure, the prompt improvement following drug withdrawal (dechallenge), and the absence of features that typically accompany malaria‐related hepatic involvement (e.g., sustained parasitemia, systemic toxicity, or need for additional antimalarial therapy). In contrast, spontaneous improvement of liver tests without further antimalarial treatment argues against malaria as the proximate cause. When viewed collectively with the patient’s clinical presentation, the cholestatic hepatitis was believed to be secondary to the antimalarial medication use rather than an active malarial hepatitis.

In summary, for patients with a recent history of malaria treatment in Cameroon who present at our hospital with severe acute hepatitis, a retrospective analysis of this case leads our team to achieve consensus on drug‐induced liver injury diagnosis. We have determined that malaria‐related hepatitis is an unlikely cause, given the patient’s clinical manifestation and spontaneous recovery without the need for additional malaria treatment.

## Consent

Patient consent to publish has been obtained.

## Conflicts of Interest

Dr. Ashwani K. Singal: GSK (Grant/Research Support), Madrigal (Speaker Bureau), Medical Speaker’s Network (Speaker Bureau), Medscape (Speaker Bureau), NIAAA (Grant/Research Support), Pleiogenix (Consultant, Stock Options), Uptodate (Royalties). The other authors declare no conflicts of interest.

## Author Contributions

All authors listed meet all criteria for authorship as per ICMJE. Chencheng Xie wrote, edited, and revised the manuscript for intellectual content and is the article guarantor and co‐corresponding author. Ashwani K. Singal edited and revised the manuscript for intellectual content and is the co‐corresponding author. Sydney Lovrien collected clinical information, wrote, edited, and revised the manuscript for intellectual content. Erin Quist edited and revised the manuscript and provided pathology images with interpretation. Dayana Maita edited and revised the manuscript for intellectual content.

## Funding

The authors received no specific funding for this work.

## Data Availability

The data that support the findings of this study are available from the corresponding authors upon reasonable request.
